# Epistatic regulation of growth in Atlantic salmon revealed: a QTL study performed on the domesticated-wild interface

**DOI:** 10.1186/s12863-020-0816-y

**Published:** 2020-02-07

**Authors:** Francois Besnier, Monica F. Solberg, Alison C. Harvey, Gary R. Carvalho, Dorte Bekkevold, Martin I. Taylor, Simon Creer, Einar E. Nielsen, Øystein Skaala, Fernando Ayllon, Geir Dahle, Kevin A. Glover

**Affiliations:** 1 Population Genetics Research group, Institute of Marine Research, P.O. Box 1870, Nordnes, NO-5817 Bergen, Norway; 2 Molecular Ecology and Fisheries Genetics Laboratory, School of Biological Sciences, Bangor University, Deiniol Road, Bangor, LL57 2UW UK; 3 Section for Marine Living Resources, National Institute of Aquatic Resources, Technical University of Denmark, Vejlsøvej 39, 8600 Silkeborg, Denmark; 4 School of Biological Sciences, University of East Anglia, Norwich, NR4 7TJ UK; 5 Sea Lice Research Centre, Department of Biology, University of Bergen, Bergen, Norway

**Keywords:** Linkage mapping, Growth, Hybrid, Introgression, Inheritance, Non-additive

## Abstract

**Background:**

Quantitative traits are typically considered to be under additive genetic control. Although there are indications that non-additive factors have the potential to contribute to trait variation, experimental demonstration remains scarce. Here, we investigated the genetic basis of growth in Atlantic salmon by exploiting the high level of genetic diversity and trait expression among domesticated, hybrid and wild populations.

**Results:**

After rearing fish in common-garden experiments under aquaculture conditions, we performed a variance component analysis in four mapping populations totaling ~ 7000 individuals from six wild, two domesticated and three F1 wild/domesticated hybrid strains. Across the four independent datasets, genome-wide significant quantitative trait loci (QTLs) associated with weight and length were detected on a total of 18 chromosomes, reflecting the polygenic nature of growth. Significant QTLs correlated with both length and weight were detected on chromosomes 2, 6 and 9 in multiple datasets. Significantly, epistatic QTLs were detected in all datasets.

**Discussion:**

The observed interactions demonstrated that the phenotypic effect of inheriting an allele deviated between half-sib families. Gene-by-gene interactions were also suggested, where the combined effect of two loci resulted in a genetic effect upon phenotypic variance, while no genetic effect was detected when the two loci were considered separately. To our knowledge, this is the first documentation of epistasis in a quantitative trait in Atlantic salmon. These novel results are of relevance for breeding programs, and for predicting the evolutionary consequences of domestication-introgression in wild populations.

## Background

The process of domestication results in a set of genetic changes as a population is taken from the wild and bred over multiple generations in captivity [see 1]. It typically consists of a mixture of selective breeding for desired traits, inadvertent selection, relaxation of natural selection, and the stochastic process of genetic drift. Traditionally, directional selection in breeding programs was practised by phenotypic selection of individuals displaying a greater than average magnitude or frequency of the trait(s) of interest, working on the premise that at least part of the trait’s variance is heritable. As genomic resources have become more accessible, the most recent developments in selective breeding have utilized the statistical correlation between genotypes and phenotypes to predict phenotypic gain in the framework of an additive genetic model. This approach is commonly referred to as genomic selection (GS), and involves analysis of genome-distributed single nucleotide polymorphisms (SNPs) on the population under selection [2, 3].

By making use of large-scale Genome Wide Association (GWAS) studies, GS is often capable of detecting loci that have low or medium contribution to the trait (< 1%) [3, 4]. GS is also more precise than traditional phenotypic selection alone due to its higher ability at quantifying Mendelian sampling across siblings. In fact, GS can result in rapid improvements of livestock and crops [5–7]. However, due to its focus on heritability (i.e., additive genetic variance) GS does not necessarily use the full complexity of the genetic architecture of polygenic traits. A possible way to improve the performance of GS would therefore be to account for non-additive genetic variation [8, 9]. Polygenic traits are often simultaneously influenced by non-additive genetic mechanisms (dominance/epistasis) [10], and while heritability is the main target of focus to improve the performance of a given breed, non-additive genetic effects may play a key role in explaining phenotypic diversity. This is especially important when looking at fitness related traits (e.g., growth, shyness, foraging, predator awareness) in populations that are subjected to domestication, but still occasionally interbreed with their wild conspecifics (e.g., Atlantic salmon, *Salmo salar* L.). Deviating selection pressures in wild and captive environments are expected to favour very different genotypes leading to distinct phenotypes in the two environments [11]. In order to understand the genetic architecture shaping the fitness of wild and domesticated fish as well as their hybrids, it is important to consider all possible types of genetic mechanisms, including dominance and epistasis.

Quantitative trait loci (QTL) mapping in experimental intercrosses represents a useful tool to provide insights into the underlying genetic basis of the variability of the investigated trait(s). By focusing on an experimental pedigree with a high degree of relatedness between individuals, QTL studies focus on loci with large to medium effects and may not only reveal the number and distribution of loci contributing to the trait of interest [12], but also elucidate potential interactions between genes affecting the phenotype [13]. Epistasis, is often ignored in quantitative trait studies [13] and breeding programs, yet has the potential to significantly contribute to the phenotypic expression of the trait [10]. The benefit of including non-additive genetic effects in genomic prediction of complex traits is however not a resolved question [14, 15].

Atlantic salmon is one of the world’s most domesticated fishes [see 11]. Commercial aquaculture, including selective breeding programs, was first initiated in Norway in the early 1970’s [16]. Increasing growth rate through both phenotypic and pedigree-based selection has been the major target for all breeding programs [17, 18]. Atlantic salmon display a high heritability in growth rate, *h*^*2*^ > 30% [18], and the genetic gain per generation has been estimated to be ~ 10–15% of the breeding values [18, 19]. Furthermore, after ~ 12 generations of directional selection, genetic gains in growth rate are still being achieved each generation [see Fig. 4 in 11]. Therefore, after nearly half a century of domestication and selective breeding, farmed salmon now out-grow wild salmon several-fold when reared together under commercial conditions [20, 21]. Nevertheless, despite large and well-documented gains in growth rate through selection, the underlying genetic mechanisms remain largely elusive.

In salmon, as in most organisms, growth is regarded as a polygenic trait [22]. The importance of additive genetic inheritance on this trait is revealed by the large heritability. Nevertheless, there are indications that growth may also be influenced by non-additive factors [23]. Several growth-linked QTLs have been documented in Atlantic salmon, and QTLs on some of the same chromosomes have been reported across studies [24–31]. However, previous QTL-studies related to growth of Atlantic salmon have been restricted to domesticated salmon, with exceptions such as the study by Baranski et al. [24] which also included a landlocked population and the study by Besnier et al. [31] that focused on domesticated/wild interactions in a natural environment.

Atlantic salmon displays several key features making it ideal to investigate additive and non-additive genetic factors on growth [32]. Firstly, fast growing domesticated salmon originate from slow-growing wild salmon [16]. The latter clearly hold the genetic potential for elevated growth rates, although this is not selected for in the wild. Second, wild Atlantic salmon display substantial population genetic structure throughout their native range [33–36], including genetic-based phenotypic and life-history variation [37]. Third, genomic resources for the Atlantic salmon are now widely available [38]. Fourth, well-established rearing systems combined with the ability to cross genetically diverse wild and domesticated salmon provides a good experimental framework in which to investigate genetic inheritance of growth. By crossing fish from unrelated multiple wild populations and domesticated strains, the chances of including allelic combinations that may reveal the genetic architecture of a complex trait such as growth, and the potential for epistasis, is increased.

**Fig. 1:**
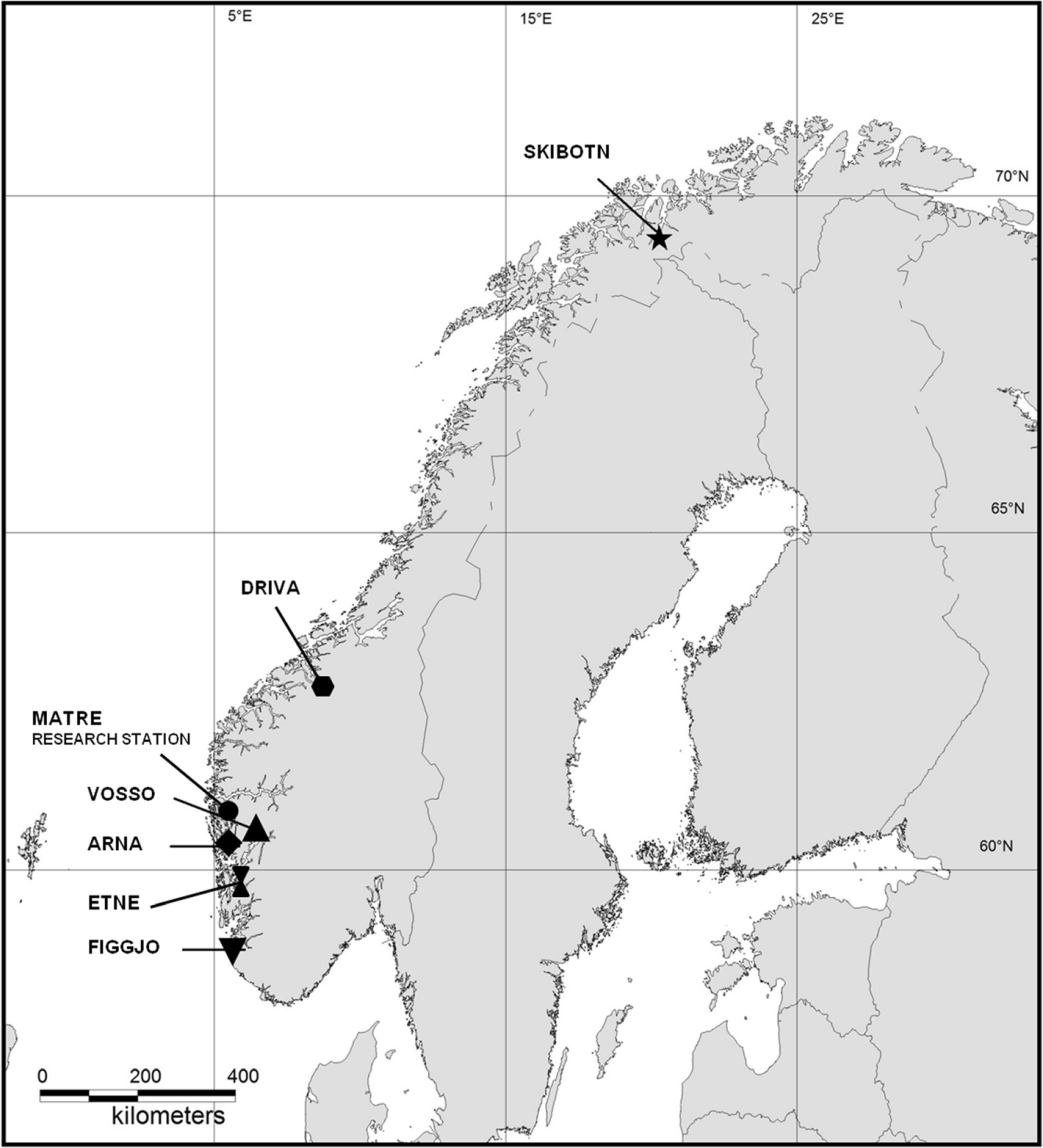
Map of wild populations and location of the Matre research station. Wild Atlantic salmon, *Salmo salar* L., populations originating from six Norwegian rivers were included in the study. Parental salmon were collected directly from the rivers, except for salmon of the River Skibotn and Driva strain that had been conserved and reared in the Norwegian Gene Bank for Atlantic salmon. Parental salmon of the River Vosso strain had been reared by the gene bank until the smolt stage, and then released into the wild. All six wild populations, two domesticated strains and three F1 wild/domesticated strains were produced and reared at the Matre research station. The genome-wide quantitative trait locus (QTL) scan for traits related to freshwater growth were performed on a total of four datasets/experiment, including 134 families and ~ 7000 individuals. The map was produced by using the software QGIS 2.8 (https://www.qgis.org/en)

In this study, we conducted genome-wide QTL mapping on multiple domesticated, hybrid and wild populations of Atlantic salmon originating from geographically distinct regions (Fig. 1). Using a variance component mapping analysis, we studied ~ 7000 individuals originating from six wild, two domesticated and three F1 wild/domesticated hybrid strains, and identified genome-wide significant QTLs for freshwater growth. By creating a mapping population of diverse genetic background, we utilized the population genetic variance observed in Norwegian Atlantic salmon. This allowed us to search for replicable QTLs in four distinct datasets representing multiple strains, indicating their relevance for several of the Norwegian salmon populations, as well as detecting evidence of non-additive genetic architecture of the quantitative trait investigated.

## Results

### Identification of QTLs within datasets

**Fig. 2:**
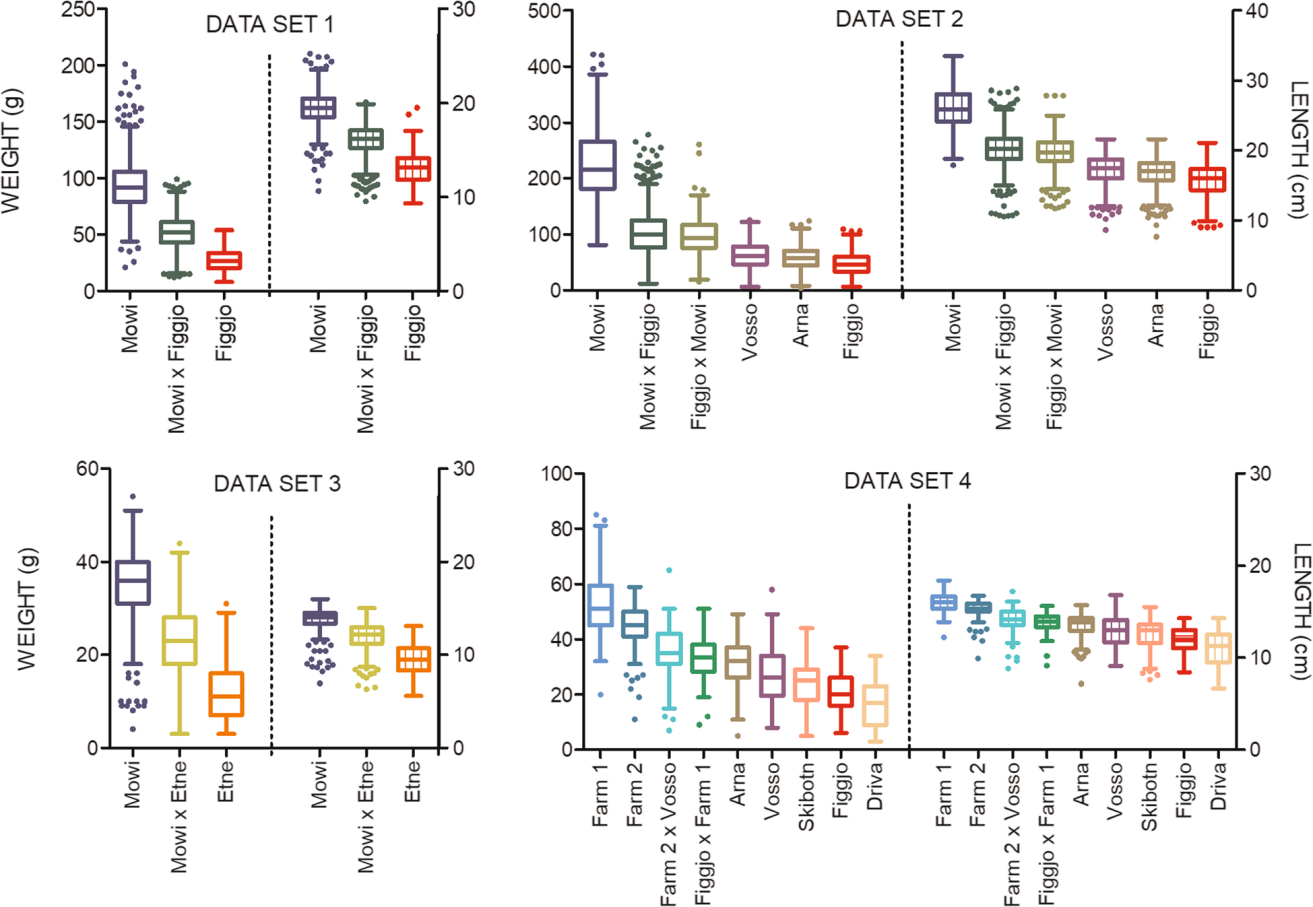
Phenotypic growth measurements of Atlantic salmon of domesticated, hybrid and wild origin. Freshwater growth of Atlantic salmon, *Salmo salar* L., of all origins, in dataset 1–4, reared communally under hatchery conditions. Weight in grams on the left y-axis, length in cm on the right y-axis. The solid line illustrates the average weight of all fish in the respective datasets, while the dotted line illustrates the average length. Error bars show standard errors. Salmon in dataset 1 and 2 were sampled after their first winter as 1+ parr/smolt, while salmon in dataset 3 and 4 were sampled after their first summer as 0+ young of the year

Genome scans for QTL detection were performed on four datasets consisting of different crosses between five wild populations and two domestic strains. The variation of fish weight and length between crosses is presented (Fig. 2).

**Fig. 3:**
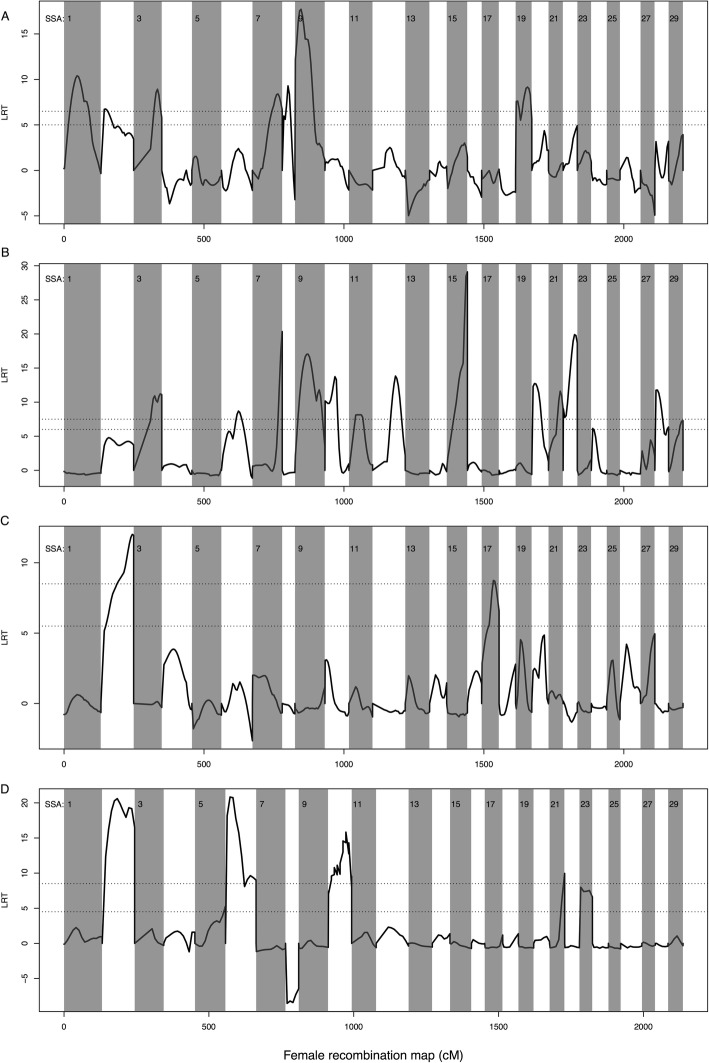
QTL scan for weight dataset 1 (3.**a**), dataset 2 (3.**b**), dataset 3 (3.**c**) and dataset 4 (3.**d**). Genome-wide scan at regular intervals (20–30 CM) of the Atlantic salmon linkage map for QTLs affecting freshwater weight, performed on the wild/domesticated interface. Horizontal lines indicate the 5 and 1% genome-wide significance threshold, based upon the likelihood ratio between a HGLM fitted at each genomic position with and without a QTL effect. Vertical lines separate chromosomes

By using similar statistical models in all datasets, i.e., not accounting for sex in dataset 1 and dataset 2, seven QTLs for weight were reported in dataset 1 on chromosomes 1, 2, 3, 7, 8, 9 and 19 (Fig. 3a), 12 QTLs in dataset 2 on chromosomes 3, 6, 7, 9, 10, 11, 12, 15, 20, 21, 22 and 28 (Fig. 3b), two QTLs in dataset 3 on chromosomes 2 and 17 (Fig. 3c), and five QTLs in dataset 4 on chromosomes 2, 6, 10, 21 and 23 (Fig. 3d) (Table 1). Each QTL accounted for 2.5–20% of the genetic variance for weight within each dataset (Table 1). By fitting all QTL effects in the same model, we estimated that the cumulated variance explained by all seven QTLs in datasets 1 to 4 accounted for 33.0, 36.2, 11.3 and 43.1%, respectively. When sex was accounted for, as a fixed covariate, the number of QTLs detected reduced to three in dataset 1 on chromosome 3, 9, and 19, and four in dataset 2, on chromosome 12, 15, 20, 22.

**Table 1:** Weight QTLs

Dataset	H2	Chromosome	Position	% explained variance	Va	Vq	Relative contribution domesticated parents	Relative contribution wild parents
1	0.22	1	20–100	6.5	35.7 ± 14.2	17.4 ± 10.1	87	13
1	0.22	2	10–40	10.4	36.8 ± 23.0	24.4 ± 14.6	71	29
1	0.22	3	60–100	2.5	47.0 ± 20.4	6.0 ± 4.0	67	33
1	0.22	7	50–100	3.1	45.8 ± 21.3	7.5 ± 4.2	70	30
1	0.22	8	5–25	12	21.0 ± 15.2	29.1 ± 12.1	82	18
1	0.22	9	5–80	9.1	31.4 ± 9.8	22.4 ± 10.6	72	28
1	0.22	19	2–60	5.4	45.9 ± 9.4	12.7 ± 6.9	85	15
2	0.19	3	60–100	3.3	198.2 ± 28.9	37.0 ± 26.8	90	10
2	0.19	6	35–45	4.1	193.2 ± 26.3	45.8 ± 25.3	82	18
2	0.19	7	80–100	5	198.2 ± 28.1	38.4 ± 25.7	87	13
2	0.19	9	5–100	3.5	197.4 ± 23.3	40.7 ± 26.9	90	10
2	0.19	10	0–50	4.8	192.4 ± 20.1	48.3 ± 29.4	90	10
2	0.19	11	30–50	5.7	193.8 ± 28.1	49.3 ± 25.1	90	10
2	0.19	12	80–110	3	201.4 ± 23.0	32.3 ± 25.7	89	11
2	0.19	15	50–75	5	192.1 ± 25.5	51.3 ± 22.1	94	6
2	0.19	20	0–30	2.7	203.23.9	27.3 ± 16.6	91	9
2	0.19	21	35–50	3.6	193.9 ± 25.9	39.8 ± 27.9	95	5
2	0.19	22	15–50	7.3	180.9 ± 22.4	64.8 ± 31.2	90	10
2	0.19	28	0–30	3.4	198.2 ± 23.9	36.1 ± 19.4	86	14
3	0.07	2	20–110	7.2	2.2 ± 0.6	1.7 ± 0.9	50	50
3	0.07	17	20–60	5.5	2.0 ± 0.8	2.1 ± 1.0	52	48
4	0.28	2	10–100	20	23.0 ± 12.5	10.6 ± 4.2	52	48
4	0.28	6	0–50	18	30.4 ± 7.3	9.1 ± 2.1	55	45
4	0.28	10	0–80	19	33.3 ± 8.5	8.1 ± 3.4	40	60
4	0.28	21	40–50	6	45.0 ± 9.8	3.2 ± 3.0	20	80
4	0.28	23	5–45	4.7	48.6 ± 8.4	2.4 ± 2.8	37	63

Chromosome, position and percentage of genotypic variation explained by the genome wide significant QTLs related to freshwater weight of domesticated, hybrid and wild salmon in dataset 1–4. The relative contribution of the parent from each type is calculated as the relative proportion of the variance of the random effects corresponding to the farm and wild parents respectively. H2 gives the heritability estimate of the given phenotype within each dataset. Va and Vq are the estimates of the polygenic and QTL variance together with their respective standard deviation

**Fig. 4:**
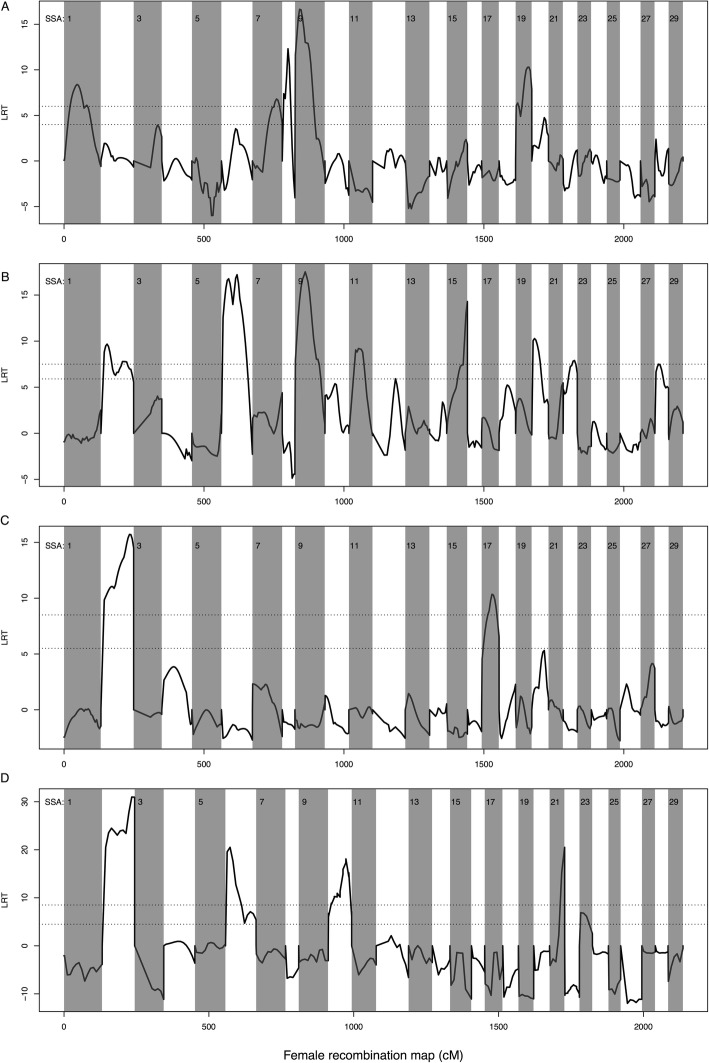
QTL scan for length dataset 1 (3.**a**), dataset 2 (3.**b**), dataset 3 (3.**c**) and dataset 4 (3.**d**). Genome-wide scan at regular intervals (20–30 CM) of the Atlantic salmon linkage map for QTLs affecting freshwater length, performed on the wild/domesticated interface. Horizontal lines indicate the 5 and 1% genome-wide significance threshold, based upon the likelihood ratio between a HGLM fitted at each genomic position with and without a QTL effect. Vertical lines separate chromosomes

Also using similar models in all datasets, six QTLs for length were reported in dataset 1 on chromosomes 1, 7, 8, 9, 19 and 20 (Fig. 4a), eight QTLs in dataset 2 on chromosomes 2, 6, 9, 11, 15, 20, 22 and 28 (Fig. 4b), two QTLs in dataset 3 on chromosomes 2 and 17 (Fig. 4c), and five QTLs in dataset 4 on chromosomes 2, 6, 10, 21 and 23 (Fig. 4d) (Table 2). Each QTL accounted for 2.8–19.3% of the genetic variance for length, within each dataset (Table 2). By fitting all QTL effects in the same model, we estimated that the cumulated variance explained by all six QTLs in datasets 1 to 4 accounted for 23.0, 33.1, 12.5 and 40.6%, respectively. When sex was accounted for as a fix covariate, the number of QTLs detected was reduced to two in dataset 1 on chromosome 9 and 19, and one in dataset 2, on chromosome 2.

**Table 2:** Length QTLs

Dataset	H2	Chromo some	Position	% explained variance	Va	Vq	Relative contribution domesticated parents	Relative contribution wild parents
1	0.12	1	20–100	6	15.2 ± 3.8	13.0 ± 3.7	68	32
1	0.12	7	50–100	7.3	16.7 ± 3.7	18.4 ± 4.2	57	43
1	0.12	8	5–25	8.7	19.4 ± 4.2	10.4 ± 3.1	60	40
1	0.12	9	5–80	9.1	13.0 ± 2.8	19.5 ± 4.0	51	49
1	0.12	19	2–60	4.6	18.2 ± 3.5	11.6 ± 4.6	64	36
1	0.12	20	33–55	4.2	21.3 ± 4.5	9.6 ± 4.4	63	67
2	0.16	2	10–100	5.2	64.2 ± 12.5	19.4 ± 12.0	70	30
2	0.16	6	30–65	10.3	50.1 ± 9.5	43.0 ± 10.2	56	44
2	0.16	9	5–100	5.1	62.0 ± 8.8	21.7 ± 9.1	74	26
2	0.16	11	25–50	7.6	58.3 ± 9.4	27.4 ± 8.9	80	20
2	0.16	15	55–75	3.8	66.1 ± 10.4	14.9 ± 7.3	82	18
2	0.16	20	5–30	2.8	68.1 ± 13.2	11.9 ± 9.6	76	24
2	0.16	22	15–50	5.7	58.2 ± 9.0	24.7 ± 9.7	79	21
2	0.16	28	5–40	2.9	67.2 ± 12.1	13.3 ± 8.8	71	29
3	0.08	2	20–110	8.3	10.1 ± 2.6	11.5 ± 3.7	32	68
3	0.08	17	20–60	6.1	10.2 ± 2.8	10.6 ± 3.1	40	60
4	0.23	2	10–100	19.3	21.7 ± 5.5	35.6 ± 4.6	28	72
4	0.23	6	0–50	15.1	17.1 ± 10.7	39.6 ± 3.9	32	68
4	0.23	10	0–80	17.2	29.9 ± 6.8	25.5 ± 5.6	25	75
4	0.23	21	40–50	13.7	36.3 ± 6.6	20.7 ± 5.2	21	79
4	0.23	23	5–45	5.5	64.8 ± 6.9	8.1 ± 6.1	27	73

Chromosome, position and percentage of genotypic variation explained by the genome wide significant QTLs related to freshwater length of domesticated, hybrid and wild salmon in dataset 1–4. The relative contribution of the parent from each type is calculated as the relative proportion of the variance of the random effects corresponding to the domesticated and wild parents respectively. H2 gives the heritability estimate of the given phenotype within each dataset. Va and Vq are the estimates of the polygenic and QTL variance together with their respective standard deviation

In datasets 1 and 2, QTL scans were performed to detect genomic regions associated with phenotypic sex. In dataset 1, chromosome 2, 3 and 6 were significantly associated with sex, whereas in dataset 2, only chromosome 2 and 6 were associated with the phenotype.

### Identification of QTLs across datasets

Several loci were consistently correlated with weight or length across datasets. QTLs located on chromosome 2 were correlated with weight in datasets 1, 3 and 4, and with length on datasets 2, 3 and 4. Similarly, QTLs on chromosome 6 were correlated with weight and length in datasets 2 and 4, while QTLs on chromosome 9 were correlated with weight and length in datasets 1 and 2. Detection of significant QTLs on the same chromosome in multiple datasets does not automatically demonstrate that the same polymorphic site is associated with the phenotypes(s) across datasets. However, between datasets, QTLs were detected at overlapping, but not always, identical positions (Tables 1 and 2).

### Family contribution to QTL

The allelic substitution effect of parental alleles was estimated separately in each family, and the relative contribution of a wild versus domestic parent was calculated for each QTL. In all datasets, both wild and domesticated parents contributed to the genome-wide significant QTLs (Tables 1 and 2). A total of 107 wild and 73 domesticated parents were used in the four datasets. On average for the four datasets, the number of domesticated parental salmon was smaller than of the wild parents. This is reflected in the ratio of domestic versus wild alleles transmitted to the offspring generation. Domestic parents accounted for 40.6% of the allelic contribution to the F1 generation, while they accounted for 73.6 and 55.0% of the relative contribution towards the genome-wide significant QTLs for weight and length respectively (Tables 1 and 2). The proportion of domestic parents contributing to the F1 generation varied between the datasets: the domesticated parents accounted for 54.1, 29.1, 52.6, and 34.0% of the F1 alleles in datasets 1–4 respectively. In comparison, the domestic parents accounted for 76.3, 89.5, 51.0 and 40.8% of the relative contribution towards the genome-wide significant QTLs for weight and 60.5, 73.5, 36.0, and 26.6% of the relative contribution towards the genome-wide significant QTLs for length.

The parental contribution to phenotypic sex was also estimated in each family separately. This per-family scan for sex determination loci indicated that a locus associated with sex determination was present on chromosome 2 for 62 and 80% of the families from datasets 1 and 2 respectively. Loci associated with sex determination was also present on chromosome 3 for 19% of the families from dataset 1 and on chromosome 6 for 19 and 20% of the families from dataset 1 and dataset 2 respectively.

### Epistasis

Two approaches were implemented to look for evidence of epistasis. First, the four datasets were screened for gene-by-gene interactions. Here, only suggestive evidence of interaction was detected which did not reach the genome-wide significant threshold. For example, in dataset 2, interaction was detected between the alleles on chromosome 20 and 24. The interaction occurred in the full sib families from parent 43 (domesticated) crossed with parent 15 (wild) and parent 36 (domesticated) where no genetic effect could be detected when considering either loci separately, while the combined effect of the two loci explained 10% of the phenotype variance in the family. Despite a nominal *p* value of 2.2.e^− 06^, the interaction between chromosomes 20 and 24 was not genome-wide significant (p ≈ 0.09) after conservative bootstrapping.

The second approach implemented to investigate epistasis was to estimate the interaction between allelic substitution effect and polygenic effect in a half-sib family context. This could only be performed when a given male was used to fertilize the egg of two distinct females (or a female having her eggs fertilized by two distinct males). In such case, genetic interaction is detectable when the allelic substitution effect changes significantly depending on which genetic background it is measured in.

**Fig. 5:**
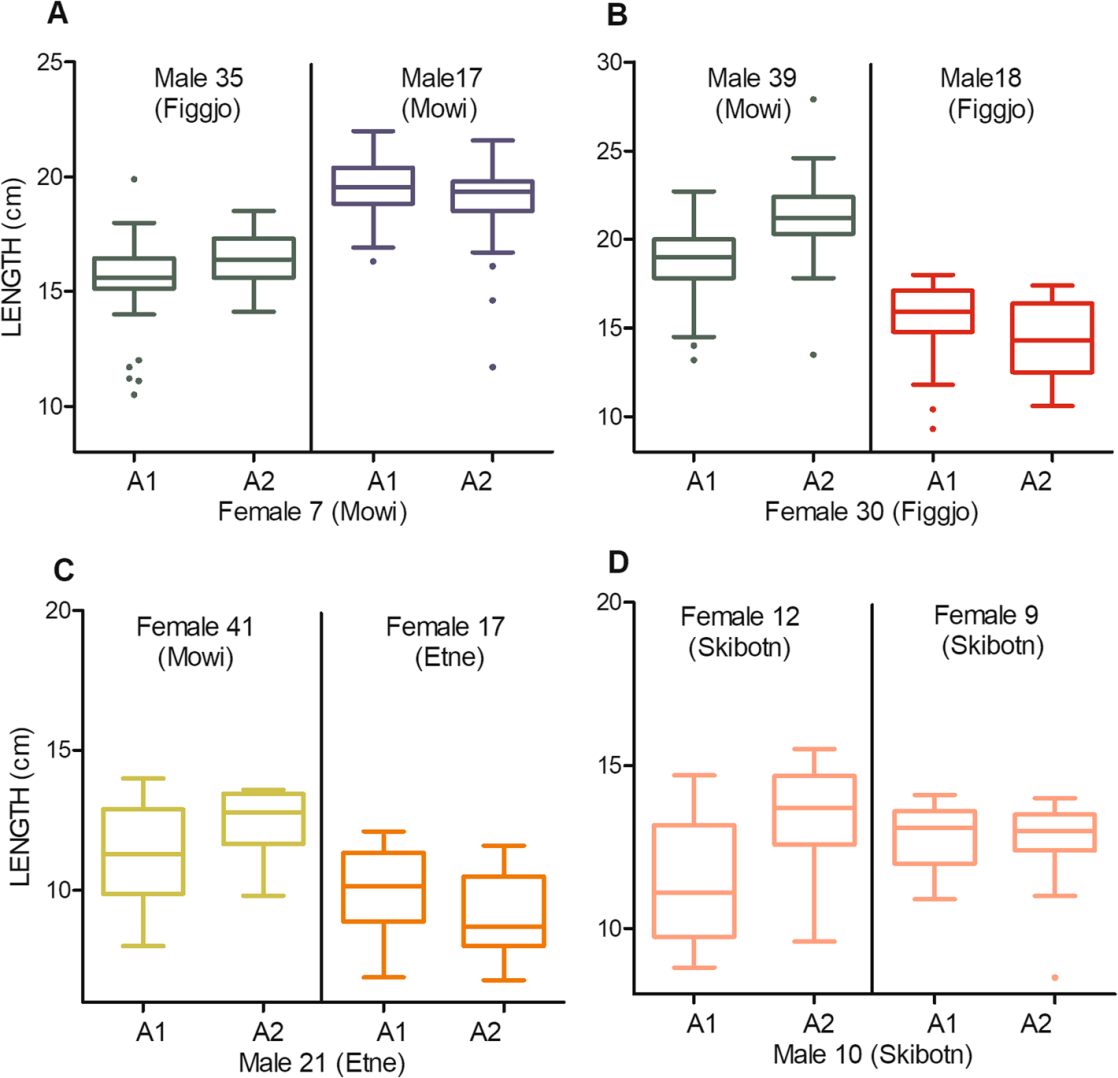
Epistatic QTLs. Phenotypic response of epistatic QTLs affecting freshwater growth in half-sibling families of Atlantic salmon. Significant gene-by-parent interactions, detected in all four datasets, demonstrate that the effect at a given allele was affected by the genetic background of the parents in the half-sib family. Selected examples from all four datasets; **a**, dataset 1; **b**, dataset 2; **c**, dataset 3 and; **d**, dataset 4

From this approach, significant epistatic QTLs for weight and length were detected in all four datasets on a total of 9 chromosomes. For example, in dataset 1, female parent 7 (domesticated) was crossed with two male parents, 35 (wild) and 17 (domesticated), to produce two half-sib families. On chromosome 17, the allelic substitution effect was significantly linked with variation in the offspring length. Notably however, the one allele inherited from female parent 7 was associated with smaller offspring in the female 7 X male 35 family, yet the same allele inherited from female parent 7 was associated with larger offspring in the female 7 X male 17 family (Fig. 5a). Therefore, the direction of the allelic substitution effect changed between the offspring of male 35 and male 17.

A similar pattern to the above example was observed in dataset 2 on chromosome 9 for offspring of female parent 30 (wild), when crossed with male parent 39 (domesticated) and 18 (wild) (Fig. 5b); in dataset 3, on chromosome 4 for offspring of male parent 21 (wild), crossed with female parent 41 (domesticated), and 17 (wild) (Fig. 5c); and in dataset 4, on chromosome 2 for male parent 10 (wild), crossed with female parent 12 (wild) and 9 (wild) (Fig. 5d).

In total, significant gene-by-parent interactions were observed in a total of 9 half-sib families, on 9 different chromosomes, across all four datasets. These gene-by-parent interactions were responsible for between 2.5 to 16.4% of the phenotype variance within these families (Table 3), thus demonstrating a significant influence of non-additive variation on the trait.

**Table 3:** Epistatic QTLs

Dataset	Phenotype	Chromosome	Position	Half-sib parent	Crossed with	% of phenotype variance within family
1	L	17	0–12	7 (Mowi ♀)	17 (Mowi ♂) and 35 (Figgjo ♂)	2.5
1	L	22	10–45	2 (Mowi ♀)	12 (Mowi ♂) and 32 (Figgjo ♂)	2.7
2	L	9	40–105	30 (Figgjo ♀)	18 (Figgjo ♂) and 39 (Mowi ♂)	5.2
2	L	28	5–50	30 (Figgjo ♀)	18 (Figgjo ♂) and 39 (Mowi ♂)	3.8
3	L	4	55–85	21 (Etne ♂)	17 (Etne ♀) and 41(Mowi ♀)	6.5
3	L	5	5–95	39 (Mowi ♀)	9 (Mowi ♂) and 32 (Etne ♂)	4.6
3	L	11	40–55	41 (Mowi ♀)	11 (Mowi ♂) and 21 (Etne ♂)	9.5
3	L	20	15–60	36 (Etne ♂)	23 (Mowi ♀), 24 (Etne ♀) and 34 (Mowi ♀)	4.2
4	L	2	15–100	10 (Skibotn ♂)	9 (Skibotn ♀) and 12 (Skibotn ♀)	9.7
4	L	17	0–40	24 (Vosso ♂)	37 (Farm2 ♀) and 23 (Vosso ♀)	14.1
1	W	9	1–55	37(Figgio♂)	9 (Mowi♀) and 29 (Figgo♀)	3
1	W	22	10–45	2 (Mowi ♀)	12 (Mowi ♂) and 32 (Figgio♂)	3.1
2	W	9	40–105	30 (Figgjo ♀)	18 (Figgjo ♂) and 39 (Mowi ♂)	5.2
2	W	28	10–45	30 (Figgjo ♀)	18 (Figgjo ♂) and 39 (Mowi ♂)	4
3	W	4	55–85	21 (Etne ♂)	17 (Etne ♀) and 41(Mowi ♀)	6.2
3	W	5	5–95	39 (Mowi ♀)	9 (Mowi ♂) and 32 (Etne ♂)	4.9
3	W	11	40–55	41 (Mowi ♀)	11 (Mowi ♂) and 21 (Etne ♂)	9.1
3	W	20	15–60	36 (Etne ♂)	23 (Mowi ♀), 24 (Etne ♀) and 34 (Mowi ♀)	4.5
4	W	2	15–100	10 (Skibotn ♂)	9 (Skibotn ♀) and 12 (Skibotn ♀)	8.6
4	W	17	0–40	24 (Vosso ♂)	37 (Farm2 ♀) and 23 (Vosso ♀)	16.4

Chromosome, position, ID of parents displaying alleles under epistatic regulation and percentage of phenotypic variation within the respective families explained by the epistatic QTL related to freshwater weight of domesticated, hybrid and wild salmon in dataset 1–4

## Discussion

In the present study, the genetic architecture underlying freshwater growth of Atlantic salmon was investigated through a genome-wide QTL scan performed on four independent datasets on the genetically divergent wild/domesticated interface. In addition to multiple QTLs, we identified multiple epistatic QTLs where the allelic substitution effect of a given locus changed depending on the genetic background it was measured in. For all epistatic QTLs, both maternally and paternally inherited, the phenotypic growth effect of inheriting a specific allele deviated between half-sib families, demonstrating that the effect of the allele was dependent on the parental genetic background. Epistasis has been defined as “the situation where the phenotype of a given genotype cannot be predicted by the sum of its component single-locus effects” [13]. Therefore, the present study provides the first experimental evidence of epistasis affecting phenotypic trait expression in one of the world’s most studied and socio-economically important fishes, the Atlantic salmon.

### Detection of QTLs and parental contribution

This study was designed to exploit the large degree of genetic divergence among domesticated and wild salmon populations [11] in order to provide knowledge regarding the genetic basis of inheritance of the investigated quantitative trait, and to identify genomic regions affecting freshwater growth of Atlantic salmon populations in general. Significant QTLs on chromosome 2, affecting freshwater growth, were detected in all four datasets. QTLs affecting growth have previously been documented on chromosome 2 in domesticated salmon of varying age, both during freshwater [27–29] and marine rearing [24–29]. The latter studies included several domesticated strains originating from both European Atlantic salmon [24–26], North American Atlantic salmon [28] as well as trans-Atlantic backcrosses [27, 29] and a landlocked population [24]. Significant QTLs on chromosome 2 affecting growth have also been documented in domesticated and wild European Atlantic salmon studied in the wild [31]. Although these results strongly suggest the presence of QTLs on chromosome 2 linked to both juvenile and adult growth in Atlantic salmon, it is worth mentioning that a QTL on chromosome 2 is not reported in all studies [30], nor at all sampling points over time, when the same mapping material is sampled repeatedly [26]. The genetic background of the mapping population, the life stage investigated or the size (growth rate) of the fish could influence deviations in the detection of significant QTLs on chromosome 2 between these studies. Deviating results among studies could also reflect gene-by-environment interactions. In fact, deviating QTLs for juvenile growth in the same mapping material of salmon families reared in two separate environments, i.e., siblings reared in a hatchery and in the wild, has been documented [39]. We also detected genome-wide significant QTLs related to weight and/or length in two or more datasets on chromosomes 3, 6, 7, 9, 10, 20 and 21. Significant or suggestive QTLs linked to growth on these chromosomes have all previously been documented across the above-mentioned QTLs studies of Atlantic salmon. Growth is a highly polygenic trait, thus the detection of significant QTLs on several chromosomes here was expected. Likewise, the four independent datasets were obtained from the crosses of different genetically divergent salmon populations. It is therefore not surprising that we observed different QTLs across datasets.

Scanning the genome also showed that salmon chromosomes 2, 3 and 6 were linked with phenotypic sex, which concurs with previous publications on sex determination in salmon [40, 41]. Phenotypic sex was included as covariate in the search for growth QTLs in datasets 1 and 2, where fish were kept long enough after termination of the experiment to reach sexual maturity and thus be phenotypically determined. In both datasets 1 and 2, the number of significant QTLs dropped strongly after including sex as covariate in the model. However, that the number of fish included in the analysis dropped from 2000 to 1170 in dataset 1 and from 2400 to 1564 in dataset 2, when including phenotypic sex, is likely to have contributed to the reduction in the number of QTLs detected (i.e., reduced statistical power). For the QTL on chromosome 6, that was both canceled by the inclusion of phenotypic sex in the model, and linked with sex determination, it is not possible to exclude the possibility that the initially detected QTL reflects the effect of sex on growth rather than an independent locus affecting growth.

In the present study, parental salmon from the two domesticated strains and the six wild populations all contributed to the significant QTLs for freshwater weight and length. In total, domesticated parents accounted for 73.6 and 55.0% of the relative contribution towards the genome-wide significant QTLs linked to body weight and length respectively, while they contributed to 40.6% of the allelic composition of the F1. However, variations in parental contribution towards significant QTLs between parents of domesticated and wild origins were detected in the four datasets. Relative to the allelic contribution, domesticated parents contributed both more and less than expected in the different datasets. Differences in their contribution towards QTLs for weight and length were also detected. Parents contributing to the epistatic QTLs were also of both wild (60%) and domesticated origin (40%). The overall result suggests low allelic fixation for the loci contributing to the growth in the domesticated parental salmon included here. Lower allelic variation at highly polymorphic microsatellites has been reported on domesticated salmon strains [42, 43]. Furthermore, in dataset 3, reduced genetic variation for growth in the domesticated salmon strain had previously been suggested due to the documentation of lower heritability estimates for this trait in the domesticated relative to wild salmon [see 44]. Here, the domesticated parents contributed to the significant QTLs for length in a lower number than they contributed with gametes, but as expected for QTLs linked to growth.

The detection of similar, even higher levels of domesticated parental contribution to the overall QTL phenotypic and genotypic variation from growth, as revealed in this study, demonstrates that selection for growth, even after more than 12 generations of directional selection for this trait, has not reached saturation. This is in agreement with results from a suite of comparative growth studies between salmon of domesticated and wild parentage across the different generation of domestication [see Fig. 4 in 11]. Thus, it appears that despite the several-fold increase of growth rate in domesticated salmon, they still contain considerable growth potential that can be utilised for further genetic gains via directional selection.

### Evidence of epistasis and its practical implications

Quantitative genetics and QTL mapping mainly focus on detecting loci that contribute additively to the phenotypic trait variation [45]. However, genetic interactions, such as epistasis and dominance, may also influence the phenotypic trait variation [13]. Here, we detected a non-additive genetic architecture of the trait investigated, i.e., growth. Notably, epistatic QTLs were observed in all four datasets included in the study. As the phenotypic growth effect of inheriting a specific allele was different between half-sib families, the parental genetic background affected the phenotypic expression of these alleles. Due to the opposing effect of inheriting a specific allele, the overall effect across the families might be evened out, and therefore a genome-wide significant QTL may or may not have been detected on that particular chromosome. We also found suggestions of gene-by-gene interactions, where the combining effect of two loci resulted in a genetic effect upon phenotypic variance, while no genetic effect was detected when the two loci were considered separately.

Non-additive inheritance in gene expressions has previously been documented [46–50]. However, and to the best of our knowledge, this is the first empirical study to reveal epistatic regulation of a quantitative trait in Atlantic salmon. Epistasis-influences on growth have been documented in other animals such as mice [10], chicken [51] and pigs [52]. The documentation of epistasis as a genetic basis for quantitative trait variation in Atlantic salmon could have implications for selective breeding programs; a topic that has been debated in both MAS [53] and GS [54]. The inclusion of non-additive effects have the potential to improve the accuracy of the predicted genetic values [54], however, the actual benefit of including non-additive genetic effects in genomic prediction is not a resolved question [14, 15].

Epistasis in domesticated, wild and hybrid half-sibling families may have implications for the long term consequences of genetic interactions between domesticated escapees and wild conspecifics [11]. Introgression of domesticated salmon has been detected in multiple wild populations in Norway and elsewhere [55–58]. In turn, this has also influenced life history traits, such as age and size at maturation [59]. Gene-flow from domesticated escapees is thought to occur mainly through domesticated females spawning with wild males [60], similar to the mating design used in dataset 1 (Fig. 5a) where we also documented epistasis. Individual-based eco-genetic models developed to study and quantify responses in wild populations to challenges such as genetic introgression, e.g., IBSEM: an individual-based Atlantic salmon population model [61, 62], are based on additive inheritance of traits. Although this approach is defendable given that additive genetic variation explains most of the genetic differences in survival in the wild between domesticated and wild salmon [31, 63–67], as well as growth under controlled conditions [20, 21, 44], the demonstrated possibility of epistasis, as revealed here, suggests that non-additive variation should not be overlooked.

### Potential limitations

The present study is based on four datasets which all consist in two generations of intercrosses between wild and domestic Salmon. While this type of experimental design is powerful to detect QTLs, it also has a number of limitations. Due to the small recombination rate in Salmon, the parents and offspring share haplotype blocks consisting of large chromosome segments. This makes it possible to follow the parent to offspring allelic transmission with only a small number of genetic markers. It also conveys very little precision regarding the actual location the causative polymorphism associated with the trait. In short, the detected QTLs often cover half a chromosome or more, and it is therefore impossible to determine whether the association between QTL and phenotype is due to one or several causative polymorphic sites. In the case of epistatic QTL, the nature of the observed interaction is not accessible either. The change of allelic substitution effect between two half-sib families can be in fact due to the change of effect of a single site, or the average change of effect between several loci.

## Conclusion

QTLs associated with weight and length were detected on 18 chromosomes, while three of these were consistent across multiple datasets, this indicates that these results are relevant for a wide range of salmon populations. Significantly, we had multiple observations that the effect of several QTL alleles changed between half-sib families, indicating epistatic regulation of growth (Table 3, Fig. 5a-d). To our knowledge, this is the first documentation of epistasis in a quantitative trait in Atlantic salmon. These novel results are of relevance for breeding programs, and for predicting the evolutionary consequences of domestication-introgression in wild populations.

## Methods

### Overall experimental design and phenotypic measurements

A genome-wide QTL scan for traits related to freshwater growth (weight and length) was performed on wild, domesticated, and F1 wild-domesticated hybrid Atlantic salmon from four independent experiments (datasets 1–4). In total, ~ 7000 salmon from 134 families, representing six wild populations, two domesticated strains and three wild-domesticated hybrid strains were included (Fig. 1). Fish within each dataset were communally-reared under standard fish farming conditions from the eyed-egg stage onwards. Growth measurements were collected from all offspring during the freshwater stage, either after their first summer as 0+ young of the year or after their first winter as 1+ parr/smolt. Prior to sampling, all individuals were anesthetised or euthanised with an overdose of metacain (Finquel® Vet, ScanVacc, Årnes, Norway). Fish were then wet weighed, fork length measured, and adipose or caudal fin clipped. Fins were preserved in 95% ethanol. All ~ 7000 individuals were originally genotyped with microsatellites markers [68–72] in order to perform parental assignment, and later with SNPs in order to link genetic variation with phenotypic variation. In addition, fish from datasets 1 and 2 were kept in the experimental facility until the adult stage, and phenotypic sex was recorded for those individuals surviving until sexual maturity. This was not performed for fish in datasets 3 or 4.

### Experimental populations

The six wild populations included in this study originate from rivers spread along the coastline of Norway (Fig. 1), encompassing both of the identified main phylogenetic groups revealed in Norway [73, 74].

For the rivers Figgjo, Arna and Etne, wild parental salmon were caught in the river, and scale samples were analysed to verify that broodfish to be used in the experiments were wild salmon and not escaped domesticated salmon from fish-farms [75]. The salmon populations in the rivers Driva and Skibotn are conserved by the Norwegian Gene bank for Atlantic salmon, and parental salmon had been reared in freshwater at Haukvik, central Norway, for between one and three generations. In the gene bank, maintenance of wild salmon and their offspring are performed without any form of directional selection, although inadvertent selection may occur. The Vosso strain is also conserved by the Norwegian Gene bank; however, at the smolt stage fish are released in the wild and only returning salmon have been used as parents in this study. For more details on the Norwegian Gene Bank program for Atlantic salmon, see [76].

The commercial Mowi strain owned by Marine Harvest AS (recently renamed Mowi AS) is the oldest Norwegian domesticated strain [16]. This strain was established in 1969 when large multi-sea winter fish were collected from the River Bolstad in the Vosso watercourse and the River Åroy, in addition to wild salmon caught at sea off Western Norway near Osterfjord and Sotra [77, 78]. Individuals from the 10th and 11th generation, i.e., both overlapping and non-overlapping year classes, were used asparents to generate the biological material (offspring) in this study. The SalmoBreed domesticated strain was commercially established in 1999 but is based upon genetic material from several Norwegian domesticated strains that have been under commercial selection since the early 1970’s. Individuals from the approximately 11th generation were used as selected parents in this study. Fin tissue samples were collected from all parental salmon to extract DNA for parental assignment and QTL-mapping.

#### Dataset 1

In 2010, adult salmon from Figgjo and Mowi were used to generate 9 families of wild, 10 families of domesticated and 10 families of F1 hybrid (domesticated ♀ x wild ♂) origin. Thus, the F1 hybrids were maternal and paternal half-siblings to the domesticated and wild salmon respectively. Two replicates of 100 individuals per family (2900 individuals/replicate) were communally reared and later split into four replicates due to increasing biomass (c. 1450 individuals /replicate). In March 2012, 500 smolt/replicate (2000 smolt in total) were sampled for growth measurements (mean ± sd; weight (g): 62.2 ± 32.3, length (cm): 16.6. ±3.0, Fig. 2). One thousand one hundred seventy of these individuals reached the adult stage within the experimental facility, and had their phenotypic sex accurately recorded. All sampled individuals were thereafter genotyped and included in this study. More information about production and rearing of these groups can be found elsewhere [21]. (Additional file 1: Table S1).

#### Dataset 2

In 2011, adult salmon from Figgjo, Arna, Vosso and Mowi were used to generate 20 families of wild, 6 families of domesticated and 15 families of F1 hybrid (reciprocal Figgjo x Mowi) origin. Thus, the F1 hybrids represented both maternal and paternal half-siblings to the domesticated and wild salmon respectively. Two replicates of 50 individuals per family (2050 individuals/replicate) were communally reared and later split into four replicates due to increasing biomass (c. 1025 individuals /replicate). In March 2013, 600 smolts per replicate (2400 smolt in total), and 71 parr were sampled for growth measurements (mean ± sd; weight (g): 103.1 ± 72.9, length (cm): 19.4 ± 4.3, Fig. 2). One thousand five hundred sixty-four of these individuals reached the adult stage within the experimental facility, and had their phenotypic sex accurately recorded. All sampled individuals were thereafter genotyped and included in this study. More information about production of these experimental populations can be found elsewhere [79]. (Additional file 1: Table S2).

#### Dataset 3

In 2009, adult salmon from Etne and Mowi were used to generate 10 families of wild, 10 families of domesticated and 9 families of hybrid (domesticated ♀ x wild ♂) origin. Thus, the F1 hybrids were maternal and paternal half-siblings to the domesticated and wild salmon respectively. Two replicates of 50 individuals per family were communally reared until September 2010, when 750 individuals/replicate were sampled for growth measurements (mean ± sd; weight (g): 22.6 ± 12.0, length (cm): 11.6. ±2.2, Fig. 2). One thousand one hundred twenty-eight of the sampled individuals (564 individuals/replicate) were genotyped and included in this study. All individuals were terminated after the experiment, therefore, phenotypic sex was not recorded. More information about production and rearing of these groups can be found elsewhere [44]. (Additional file 1: Table S3).

#### Dataset 4

In 2013, adult salmon from Figgjo, Arna, Vosso, Driva, Skibotn, Mowi and SalmoBreed were used to generate 19 families of wild, 8 families of domesticated and 8 families of F1 hybrid origin. Both domesticated strains will from here on be referred to as Farm1 and Farm2 (random order). In total, two wild-domesticated F1 hybrid strains were produced by crossing the wild Figgjo and Vosso population with the two domesticated strains. Thus, the F1 hybrids were paternal and maternal half-siblings to one of the domesticated strains and the Figgjo strain, or maternal and paternal half-siblings to one of the domesticated strains and the wild Vosso strain respectively. Two replicates of 30 individuals per family (1040 individuals/replicate) were communally reared until September 2014 when 700 fry per replicate were sampled for growth measurements (mean ± sd; weight (g): 32.4 ± 13.5, length (cm): 13.5 ± 1.9, Fig. 2). All sampled individuals were genotyped and included in this study. All individuals were terminated after the experiment, therefore, phenotypic sex was not recorded. More information about production and rearing of these groups can be found elsewhere [20]. (Additional file 1: Table S4).

### Genotyping and parentage assignment

For the QTL analysis of fish from all four datasets described above, an initial set of 116 genome-wide SNP markers were selected. These were selected from the genome to optimize genomic information content for QTL mapping and was based on both the marker position on the salmon genome and allelic frequencies in the parental generation. SNPs were selected for providing genotype information at regular intervals of 20–30 cm in the female recombination map, and for being polymorphic within full sib families. Where possible, SNPs displaying heterozygous parental genotypes in each family were prioratised. Previously, these SNPs have been shown to provide the best information content using some of the same strains and populations as in the present study [31]. SNP genotyping was performed on a MassARRAY Analyzer 4 from Agena Bioscience™, according to the manufacturer’s instructions. From the initial 116 SNPs, we produced 4 multiplexes containing a total of 114 SNPs (32, 32, 29 and 21 SNPs/ multiplex). A limited number of the SNPs did not amplify, leaving the final mapping dataset to include 109 genome-wide distributed SNPs.

Parentage testing of offspring from all four datasets was initially performed using FAP v3.6 [80], and six microsatellites. Following the exclusion-based approach implemented in FAP, 97 to 99% of the offspring were unambiguously assigned to their family of origin. After SNP genotyping, the microsatellite parental assignment was double-checked using the 109 SNP markers and a custom R script [R Core Team 81]. No discrepancies in parentage assignment were detected between the marker classes, and all fish not unambiguously assigned using microsatellites were subsequently assigned using SNPs. Therefore, all individuals were used in the analysis.

### QTL mapping

#### Variance components

In each of the four datasets, the mapping population consisted of two generations (parents and offspring) from a total of 29 to 41 full and half-sib families. In each family, each offspring allele originated from one of the four parental haplotypes: two maternal and two paternal haplotypes. In order to perform QTL mapping, we first reconstructed the haplotypes of both parents and offspring based on pedigree and genotype data [82]. Compared to raw genotype data, the haplotyped data contains additional information regarding the parental (maternal or paternal) origin of each offspring allele, and identifies parental alleles linked within the same gametic haplotype. The next step consisted of estimating the Identity By Descent (IBD) coefficient between each pair of individuals at each locus along the genome. IBD coefficients were obtained from a recursive approach [83] implemented to account for haplotype information as input. The IBD coefficients are therefore estimated from the combined information at several markers. The QTL scan was thereafter performed by fitting a Mixed Linear Model at each genomic location as:

1 $$ y= X\beta + Ga+ Zq+e $$

where *y* is the phenotype vector, *X* the design matrix for fixed effects, β the vector of fixed effects, *G* the kinship matrix, *a* the vector of normal-distributed random polygenic effects, *Z* the design matrix for allelic effects, *q* the vector of normal-distributed random QTL effects, and *e* the normal-distributed random residuals. Note that GG’ is equivalent to the square kinship matrix, and covariance structure for the random polygenic effects, and ZZ’ is equivalent to the square IBD matrix and covariance structure of the random QTL effects. The fixed part of the model (*Xβ*) consisted of the effect of strain and replicate. When phenotypic sex was available, sex was also incorporated in the model as fixed covariate. The Mixed model was fitted with the R package HGLM [84].

At each tested genomic position, the likelihood of model 1 is compared to the likelihood of the model without QTL effect:

2 $$ y= X\beta + Ga+e $$

In both models, we consider the adjusted profile log-likelihood profiled over random effects as provided by HGLM [84]. The likelihood ratio between model 1 and model 2 is then considered as the indicator for QTL i.e., correlation between genotype and phenotype variance. To account for multiple testing along the genome, the genome wide significance threshold for likelihood ratio was obtain through permutation test as in GA Churchill and RW Doerge [85].

After the initial genome scan, all significant QTL were fitted into the same model (model3) in order to estimate the proportion of genetic variance explained by all QTL simultaneously.

3 $$ y= X\beta + Ga+{\sum}_{i=1}^n{Z}_i{q}_i+e $$

Where Z_*i*_ is the design matrix of QTL effect at QTL *i*, q_*i*_ the vector of random QTL effects at QTL *i,* and n is the number of significant QTL detected in the dataset.

#### Family-based fixed effect model

To identify the parental alleles associated with phenotypic variation, a simpler linear model was fitted with fixed genetic effects. The model was applied on the offspring of each parent separately:

4 $$ y= Zq+e $$

where z is a two-column design matrix indicating for each offspring, the probability of having inherited either of the two parental alleles at a given locus, q is the allelic substitution effect at each locus i.e., the average difference between the phenotype of the offspring that inherit allele 1 or allele 2 from the same parent. When a given parent had offspring with two different mates or more, a fixed mate affect was added to the model, as well as an interaction term between the allelic substitution effect and the mate effect, as follows:

5 $$ y= X\beta + Zq+ ZXp+e $$

where X is the design matrix for the mate effect a, β the mate effect, and p the interaction term.

#### Epistasis

Investigation of non-additive genetic effects was also performed in order to detect possible gene-by-gene or gene-by-parent interactions. In the case of gene-by-gene interaction, a two loci model was fitted for each locus pair across the genome:

6 $$ y= X\beta +{Z}_1q+{Z}_2p+e $$

7 $$ y= X\beta +{Z}_1q+{Z}_2p+{Z}_1{Z}_2r+e $$

where q and p are the allelic effects at locus 1 and locus 2 respectively, and Z_1_ and Z_2_ are the corresponding design matrices, and r is the vector of the interaction terms between effects of locus 1 and locus 2. The magnitude of the interaction effect between locus 1 and locus 2 was tested by likelihood ratio between model 5 and model 6. In a similar fashion, interactions between parents and genotypes were tested by comparing the likelihood of model 5 and model 8 that include an interaction term between allelic effect and mate:

8 $$ y= X\beta + Zq+ XZr+e $$

## Supplementary information

**Additional file 1:** **Table S1-S4.** Four datasets analyzed in this work. Supplementary tables are formatted as semi-column separated text files. For each dataset, the table contains the individual ID in column1, the parental IDs in column 2 and 3, the family ID in column 4, the type of cross in column 5, length and weight in column 6 and 7. The following columns contain the SNP genotype for each individual.

## Data Availability

All data analysed during this study are included in this published article as supplementary files (File S1 to S4).

## Abbreviations

GS: Genomic Selection
GWAS: Genome Wide Association Study
HGLM: Hierarchical Generalized Linear Model
MAS: Marker Assisted Selection
QTL: Quantitative Trait Locus (loci)
SNP: Single Nucleotide Polymorphism
